# Notch2 Signaling Sensitizes Endothelial Cells to Apoptosis by Negatively Regulating the Key Protective Molecule Survivin

**DOI:** 10.1371/journal.pone.0008244

**Published:** 2009-12-11

**Authors:** Thibaut Quillard, Julie Devalliere, Mathias Chatelais, Flora Coulon, Céline Séveno, Mathilde Romagnoli, Sophie Barillé Nion, Béatrice Charreau

**Affiliations:** 1 INSERM, UMR643, Nantes, France; 2 CHU Nantes, Institut de Transplantation et de Recherche en Transplantation, ITERT, Nantes, France; 3 Université de Nantes, Faculté de Médecine, Nantes, France; 4 INSERM, UMR 892, Nantes, France; Karolinska Institutet, Sweden

## Abstract

**Background:**

Notch signaling pathway controls key functions in vascular and endothelial cells (ECs) where Notch4 plays a major role. However, little is known about the contribution of other Notch receptors. This study investigated regulation of Notch2 and further examined its implication in EC dysfunction.

**Methodology/Principal Findings:**

Here, we provide evidence for a novel link between Notch and TNF signaling, where Notch2 is upregulated and activated in response to TNF. Forced expression of Notch2 intracellular domain in cultured ECs promotes apoptosis and allows the significant downregulation of several cell-death-related transcripts in a dose-dependent manner. In particular, activation of Notch2 led to a rapid decrease in survivin mRNA and protein expression, while survivin upregulation was obtained by the selective knockdown of Notch2 in ECs, indicating that survivin expression is controlled at the Notch level. Moreover, Notch2 silencing and ectopic expression of survivin, but not XIAP or Bcl2, rescued ECs from TNF and Notch2-mediated apoptosis, respectively.

**Conclusions/Significance:**

In conclusion, TNF signaling activates Notch2 that sensitizes ECs to apoptosis via modulation of the key apoptosis regulator survivin. Overall, our findings also indicate that specific Notch receptors control distinct functions in vascular cells and inflammatory cytokines contribute to this specificity.

## Introduction

Notch signaling pathway regulates a broad array of cell fate decisions in various tissues and in all stages of development (embryonic to adult). The Notch family comprises heterodimer transmembrane receptors consisting of an extracellular domain and a noncovalently linked intracellular domain (ICD). In mammals, 4 Notch receptors (Notch1-4) and 5 ligands (Delta-like [Dll]-1, Dll3, Dll4, Jagged1 and Jagged2) have been identified [1]. Upon interaction with ligands on neighbouring cells, Notch undergoes proteolytic cleavages managed sequentially by ADAM proteins (a desintegrin and metalloproteinase) and the γ-secretase complex. Release of the cytoplasmic Notch C-terminal intracellular domain (NICD) from the plasma membrane is followed by its translocation into the nucleus where it forms a complex with CSL, removing the repression and allowing for target genes (*hes, hey*) transcription [2].

Notch proteins display a selective cellular and tissue distribution. The vascular cells express all four Notch receptors but only Notch4 displays an almost exclusively endothelial expression pattern whereas Notch1-3 are expressed more ubiquitously [3]. In the vasculature, Notch1 and Notch4 are predominantly endothelial, prominent in both arteries and veins while the expression of Notch2 has been reported in pulmonary endothelium [4]. Notch3 is primarily expressed in adult arterial vascular smooth muscle cells (VSMCs). In addition to a specific pattern of tissue expression, several observations suggest that temporal regulation of Notch signaling may be equally important to control the diverse functions of the Notch family. Notch signaling plays a critical role in vascular development and homeostasis [5] and is involved in vasculogenesis, angiogenesis, differentiation, vascular remodeling, and maturation [6]. Combined deletion of *Notch1* and *Notch4* genes enhances the defects in vasculature remodeling observed in Notch1 single knock-out mice [7]. Endothelial cell (EC)-specific expression of an activated form of Notch4 leads to embryonic lethality with abnormal vessel structure and patterning [8]. Constitutive activation of Notch4 in ECs also causes defects in vascular remodeling [8]. *In vitro* experiments also demonstrate that Notch4 activation protects ECs from apoptosis, promotes endothelial-to-mesenchymal trans-differentiation and blocks both proliferation and angiogenesis [9], [10].

Endothelial cells control vascular tone, leukocyte adhesion and thrombosis by fine-tuned regulation of many cell surface and soluble molecules [11]. EC activation is considered to be an early event which subsequently leads to EC dysfunction and ultimately to vascular injury, key events associated with acute and chronic inflammation, such as occurs during sepsis, atherosclerosis and acute vascular and chronic allograft rejection. Tumor necrosis factor (TNF), an important mediator of innate inflammation, acts on vascular ECs to promote the inflammatory response. In cultured human ECs, human TNF causes apoptosis, especially in the presence of RNA or protein synthesis inhibitors such as actinomycin D or cycloheximide (CHX), respectively. TNF activates both NF*κ*B and AP-1 in ECs, leading to the expression of pro-inflammatory proteins, such as E-selectin (CD62E), ICAM-1 (CD54), VCAM-1 (CD106) and IL-8. TNF also promotes expression of an array of “protective” genes including the zinc finger protein A20, heme-oxygenase-1 (HO-1) and Bcl-xL [12]. It has been suggested that Notch is necessary for the establishment and/or maintenance of quiescent EC phenotype [10]. However, a role for Notch signaling in activated EC phenotype and function upon inflammation has not been documented.

In a previous study, we showed that impaired Notch4 expression caused by pro-inflammatory cytokines in cardiac allograft vessels promotes EC dysfunction and transplant arteriosclerosis [13]. Although the importance of Notch4 in controlling EC proliferation, differentiation and survival has been established, little is known about the role of Notch2 expressed on vascular endothelium. This study further investigates the regulatory crosstalk between TNF signaling and Notch receptors expression and activity in primary cultures of human vascular ECs. In particular, we show that TNF strongly upregulates Notch2 on vascular ECs. Moreover, the present work establishes the direct contribution of Notch2 signaling in the transcriptional regulation of several pro- and anti-apoptotic molecules. Both forced Notch2 NICD (N2ICD) expression and Notch2 silencing demonstrate interplay between Notch2 signaling and survivin expression in the control of EC apoptosis. Taken together, our findings indicate that dysregulated Notch2 signaling by TNF sensitizes vascular endothelial cells to apoptosis by the downregulation of a set of mediators of apoptosis and suggest a major role for survivin as effector of Notch signaling.

## Materials and Methods

### Ethics Statement

Informed written consent was obtained from patients. The study was performed according to the guidelines of the local ethics committee (Comité Consultatif de Protection des Personnes dans la Recherche Biomédicale [CCPRB], CHU de Nantes, France).

### Cell Culture and Reagents

Primary ECs cultures from human artery (HAEC) and umbilical vein (HUVEC) were isolated and grown in early passages (2–6) as we previously reported[14]. Human aortic endothelial cells (HAECs) were isolated from unused aortic pieces collected at the time of kidney transplantation and harvested according to good medical practice and stored in the DIVAT Biocollection (French Health Minister Project no. 02G55). HUVEC were only used for plasmid transfection experiments. ECs were cultured in endothelial basal growth medium (ECBM, Promocell, Heidelberg, Germany) supplemented with 10% fetal calf serum (FCS), 0.4% EC growth supplement/heparin, human epidermal growth factor (0.1 ng/mL), human basic fibroblast growth factor (1 ng/mL), hydrocortisone (1 µg/mL), gentamicin (50 µg/mL), and amphotericin (50 ng/mL). Before activation, confluent EC monolayers were maintained for 24 h in basal ECBM supplemented with 2% FCS and then incubated with Human Recombinant TNF (100 U/mL, provided by Professor P. Neuman, BASF, Ludwigshafen, Germany). To induce EC apoptosis, ECs were pre-treated 1 h with 100 µM pyrrolidine dithiocarbamate (PDTC, Sigma–Aldrich, St. Louis, MO, USA) before TNF addition and incubation for 24 h. To protect ECs from apoptosis, AdN2ICD-transduced cells were cultured for 48 h in presence of zvad (R&D systems).

### DNA Constructs, Small Interfering RNAs and Transfections

The luciferase reporter plasmid that contains 4 copies of a binding site for CBF1 (CBF1-Luc) was a kind gift from Dr. Diane Hayward (Johns Hopkins University, Baltimore, Maryland, USA)[15]. Cells were treated with DAPT (10 µM, N-[N-(3,5-Difluorophenacetyl)-L-alanyl]-Sphenylglycine t-butyl ester, Sigma–Aldrich) or co-transfected with the N2IC/MSCV plasmid encoding Notch2 NICD as a positive control for Notch activity[16]. Gene reporter activity was measured with the luciferase assay (Promega, Madison, WI, USA) and expressed as a relative luciferase activity after normalization to protein content. HUVEC were transfected at 70–90% confluence using the Lipofectamine and the PLUS reagent (Invitrogen, Carlsbad, CA, USA) for 1 h at 37°C in DMEM. Survivin, Bcl2 and XIAP full cDNAs were cloned into pcDNA3.1 vector. For gene silencing, 10^5^ HAECs were transfected in 6-well plates with RNAiMax lipofectamine® (Invitrogen) and siRNA targeting Notch2 (ID#144339, 72% knockdown, ID#144349, 78% knockdown, ID#144373) or a scrambled negative control (#AM4611) at a final concentration of 10 nM (Ambion, Austin, TX, USA). High transfection efficiency was verified with fluorescent siRNA (over 85%) (fluorescent Block-IT®, invitrogen). Validation of siRNA and functional assays were performed 48 h post-transfection. For each experiment, specific expression knockdown (>70%) was established by qRT-PCR.

### Generation of a Recombinant Adenoviral Vector Encoding Notch2ICD

The N2IC/MSCV plasmid encoding Notch2 NICD was kindly provided by Dr Christopher A. Klug CA (University of Alabama at Birmingham, AL, USA). The 2.3-kbp EcoRI fragment from N2IC/MSCV, containing the coding region of Notch2 intracellular domain was subcloned into pT/BH vector. The EcoRV-NotI fragment was then inserted under the cytomegalovirus promoter into the vector pTrackCMV that contains a second expression cassette for GFP dependant on CMV promoter. The resultant pTrackCMV-N2ICD vector was tested in HUVEC transfections before adenovirus generation. The recombinant adenovirus AdN2ICD was produced in the human embryonic kidney 293 cells by the vector core laboratory of the University Hospital of Nantes (INSERM UMR 649 Gene Therapy Laboratory, Nantes, France) as we previously described[17]. The recombinant adenoviruses AdTrack-GFP and –AdNull (Ad Dl324) without GFP cDNA were used as a control (AdGFP, AdNull). Efficiency and adenoviral infection and GFP expression are shown in the supplemental Figure S1. When applicable, dose-reponse of multiplicity of infection (moi) were performed to confirm/validate our findings. HAEC or HUVEC were cultured 70–90% confluence and infected with a moi of 30/cell or 300 for AdN2ICD and AdNull and 5 moi or 50 for AdGFP, respectively. Adenoviral infection was carried out in ECGM supplemented with 1% FCS for 3 h at 37°C, 5% CO_2_ under agitation. The cells were washed with medium containing 10% FCS and grown in fresh supplemented endothelial cell growth medium. Transduction efficiency was analyzed 24 h after infection through GFP detection by direct microscopy imaging and Flow Cytometry using a FACScalibur® (BD Biosciences, Franklin Lakes, NJ, USA).

### Reverse Transcription-Polymerase Chain Reaction RT-PCR & PCR Arrays

RNA was isolated using Trizol reagent (Invitrogen) and treated with Turbo DNase® (Ambion) before reverse transcription (RT). Subsequent to RT, cDNAs were amplified by PCR and analyzed in agarose gels stained with ethidium bromide. Quantitative PCRs were performed using the ABI PRISM 7700 and 7900 sequence detection application program (PE Applied Biosystems, Foster City, CA, USA). For quantification, duplicates were normalized by the concomitant quantification of hypoxanthine-guanine phosphoribosyl transferase (HPRT). Relative expression was calculated according to the 2^−ΔΔCt^ method, as previously described[18]. Primers for Notch1 (5′-GACGGACCCAACACTTACAC-3′;5′-TCAGGCAGAAGCAGAGGTAG-3′), Notch2 (5′-GCAGGAGGTGGATGTGTTAG-3′;5′-CCAGGATCAGGGGTGTAGAG-3′), Notch3 (5′-CTCATCCGAAACCGCTCTAC-3′;5′-AGTCTCTCCTGGGCTACGTC-3′), Notch4 (5′-TGTTTGATGGCTACGACTGT-3′;5′-TCCTTACCCAGAGTCCTACC-3′), hey1 (5′-CAGGCAACAGGGGGTAAAGG-3′;5′-GTGGAGCGGATGATGGTGGT-3′) and β-actin (5′-TCTGGCACCACACCTTCTAC-3′;5′ CAGCTTCTCCTTAATGTCAC-3′) were obtained from MWG (High Point, NC, USA) and used for RT-PCR analysis. Human Apoptosis PCR array primer sets (Real Time Primers, Elkins Park, PA, USA) were used at a 0.3 µM final concentration. Transcript levels were quantified by qRT-PCR with the following primers and probe from Applied Biosytems: Notch2 (Hs00225747_m1), hey1 (Hs00232618_m1), survivin (Hs00977611_g1), bim (Hs00197982_m1), DAPK2 (Hs00204888_m1), HRK (Hs00705213_s1), DR5 (Hs00366272?>_m1), CD40 (Hs00374176_m1), APRIL (Hs00182565_m1) and HPRT (H99999909_m1).

### Western Blot Analysis

Cells were lysed on ice in RIPA lysis buffer supplemented with Protease Inhibitors Cocktail (PIC, Sigma–Aldrich). Cell lysates were resolved by SDS-PAGE (7.5%–10%) and subjected to Western immunoblot using specific antibodies for Notch2 (DSHB, Iowa City, IA, USA), cleaved-Notch2 (Millipore, Temecula, CA, USA) survivin (R&D Systems, Abingdon, UK), VCAM-1 (Santa Cruz Biotechnology), and GAPDH (Oncogene, MERCK EuroLab, Val de Fontenay, France) and secondary horseradish peroxidase-labeled antibodies (CST, St Quentin-en-Yveline, France; Serotec, Martinsried, Germany). Antibody-bound proteins were detected using an enhanced chemiluminescence kit (ECL, Amersham, Buckinghamshire, UK). Results shown are representative of at least 3 independent experiments.

For Immunofluorescence, cytospins of ECs transfected with pTrackCMV-N2ICD were subjected to Notch2 labelling with Notch2 antibody (DSHB).

### Cell Viability and Apoptosis Analysis

#### DNA content

Cellular DNA content analyses were performed by flow cytometry as follows: 48 h after transfection, ECs were harvested using trypsin/EDTA, washed twice in PBS, fixed in ice-cold 70% ethanol under vortexing, and incubated for 24 h at 4°C. Fixed cells were then stained with 50 µg/mL propidium iodide (PI), 100 µg/mL RNase A (Sigma-Aldrich) in PBS (1 ml/1×10^6^ cells). For specific apoptosis detection, ECs were harvested, washed and resuspended in 100 µl of Binding Buffer (10 mM HEPES, pH 7.4, 140 mM NaCl, 2.5 mM CaCl_2_) with 5 µl of annexin V-APC (BD Biosciences, Franklin Lakes, NJ, USA) for 15 min and with 50 µl/mL of PI for 5 min. Fluorescence was measured on 10,000 cells/sample using a LSR II® (BD Biosciences) and analyzed using FlowJo® software (Tree Star, Inc.). Results shown are representative of at least 3 independent experiments.

#### Measurement of caspase activity

Caspases-3 and -7 activity was assessed using the Caspase-Glo® 3/7 Assay, according to the manufacturer's recommendation (Promega, Charbonnieres, France). Briefly, 10 µg of cell lysate were incubated with caspase substrate and fluorescence was analyzed for 2 h with a Fluoroskan Ascent™(Thermo Scientific, Saint-Herblain, France).

Caspase activity was also analyzed by western blot using antibodies against Cleaved Caspase-3 (Asp175), Cleaved Caspase-6 (Asp162), Cleaved Caspase-7 (Asp198), Cleaved Caspase-9 (Asp315), Cleaved Caspase-9 (Asp330) and Cleaved PARP (Asp214) from CST and antibody against total caspase-3 (Santa Cruz Biotechnology, CA, USA).

The cell-permeable fluorigenic peptidic substrate PhiPhiLux-G2D2 (OncoImmunin, Gaithersburg, MD) containing the cleavage site DEVD was used to monitor caspase-3-like activity in intact cells. Cells were incubated with the substrate solution for 1 h at 37°C in the dark, according to the manufacturer's instructions. Caspase 3 activation/apoptosis was followed during 18 h by time lapse imaging using a microscope DMI6000B (Leica Microsystèmes SAS. Rueil Malmaison) equipped with an objective lens X40 (HCX FL Plan), and a CCD camera (Coolsnap HQ2, Photometrics Roper Scientifics SAS Evry). Caspase 3 positive cells/fied were counted every 2 h between 48 h and 66 h post-infection with AdN2ICD and controls adenovirus. Results were expressed as the percentage of caspase-postive ECs.

### Cell Proliferation

Cells were seeded at 25% of confluence, treated with FGF-2 (R&D Systems) and pulsed for 16 h with 0.5 µCi/well [methyl-^3^H] thymidine (Amersham, Les Ulis, France). Thymidine incorporation was measured using a scintillant counter (Top count NXT, Perkin Elmer, Waltham, Massachussetts, MA, USA).

### Statistics

Data are represented as means±SEM for replicates experiments. Statistical analysis was performed with Graphpad Prism® Software (Graphpad Software, San Diego, CA) by the parametric analysis of variance test as appropriate. p<0.05 was considered statistically significant.

## Results

### TNF Induces Notch2 Signaling in Vascular ECs

**Figure 1:**
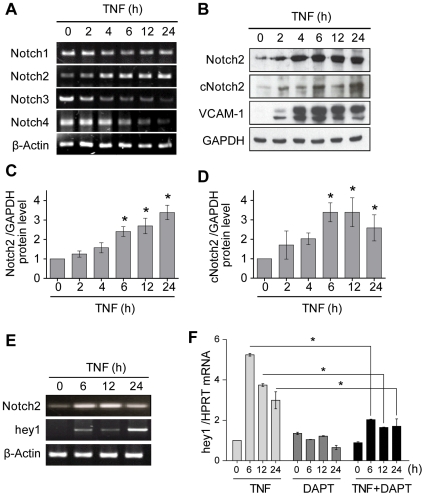
Regulation of Notch2 signaling in response to TNF. (**A**) RT-PCR for Notch1-4 in ECs treated with TNF. A western blotting analysis (**B**) and its quantification showing the expression of the native Notch2 (**C**) and the activated cleaved form (cNotch2) in response to TNF (**D**). (**E**) RT-PCR for Notch2 and hey1 in ECs treated with TNF. (**F**) qRT-PCR for hey1 in EC stimulated with TNF and DAPT. *p<0.05 *versus* controls.

TNF in human vascular ECs activates signaling pathways that regulate coordinately the transcription of a large set of molecules involved in inflammation, coagulation, thrombosis, vascular tone, immune response [11]. Moreover, TNF signaling also regulates a fine tune balance of pro- and anti-apoptotic factors that control EC survival and, ultimately, vascular injury and remodeling [19]. Consequently, the first aim of this study was to investigate how TNF signaling may control the expression of Notch receptors (Notch1, 2, 3 and 4) in cytokine-activated ECs. Firstly, primary cultures of ECs were treated for 0 to 24 h with recombinant TNF and transcript levels were analyzed by RT-PCR. As shown in Figure 1A, TNF selectively modulates the mRNA steady-state levels of the Notch receptors. TNF decreases transcript levels for Notch1, Notch3 and Notch4 with a significant effect starting 2 h after treatment and with a maximal inhibition at 24 h of 49±7%, 88±2% and 78±2% for Notch1, 3 and 4 as compared with basal levels (p<0.05 for all). In contrast, an enhanced mRNA level for Notch2 was found in response to TNF, corresponding to a 3.3±0.3-fold increase at 24 h (p<0.05) as compared with the basal mRNA level. TNF-regulated expression for Notch2 was further confirmed by western blotting (Figure 1B–D). TNF also triggers the activation of Notch2 signaling as reflected by the induction of the activated cleaved-form of Notch2 receptor (c-Notch2) (Figure 1B). Expression of, Hairy/Enhancer of split (hes) and Hairy-related (hey) transcription factors has been shown to be dependent on Notch receptors activation [2]. Among these molecular targets of Notch activity, TNF was found to drive a selective increase in hey1 mRNA (Figure 1E) while, in contrary, hes1 and hey2 mRNA exhibit a significant decrease (data not shown). Activation of Notch signaling in response to TNF, reflected by hey1 transcription, measure by qRT-PCR, was strongly abrogated in the presence of a γ-secretase inhibitor (DAPT) (Figure 1F). Together, these findings indicate that TNF strongly modulates the pattern of Notch receptors expressed in ECs by increasing Notch2 expression and activity in parallel to the induction of hey1 effector gene.

### Notch2 Signaling Elicits EC Death and Apoptosis

Despite the strong implication of Notch in endothelial and vascular biology, there have been few studies addressing the role of Notch2 in vascular cell phenotype and function. Our analysis thus focused on regulating the Notch2 pathway in human vascular ECs. To this end, we first generated a recombinant adenoviral vector encoding the intra-cellular domain of Notch2 (N2ICD) and GFP as a reporter gene (AdN2ICD). Controls were ECs transduced with a recombinant adenovirus encoding GFP alone and empty vector (AdGFP and AdNull) to account for any effects that may be due to adenoviral infection or/and GFP expression. Western blot analysis further indicates that, N2ICD expression is dose-dependent in transduced ECs (supplemental Figure S1C). Validation of the N2ICD transgene was also attested by its nuclear translocation as well as its ability to promote canonical Notch pathway activity (supplemental Figure S1D-E).

**Figure 2:**
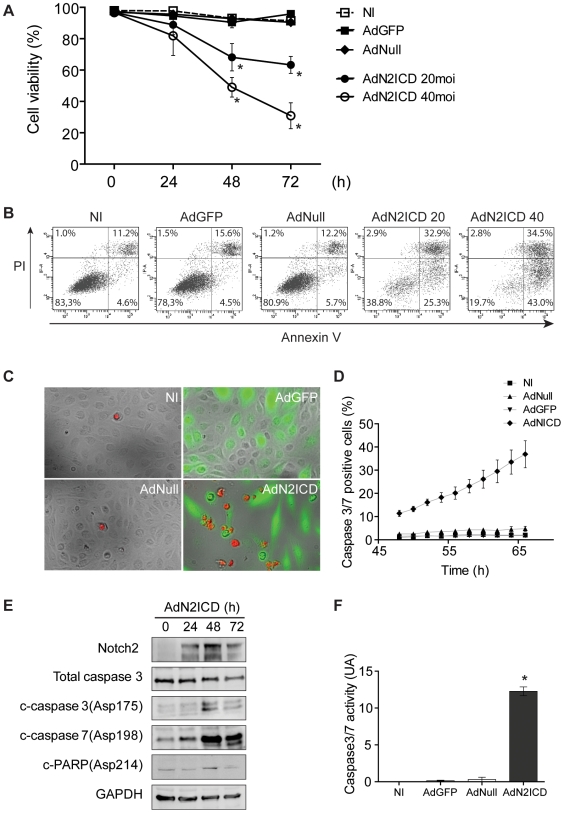
Notch2 activation elicits EC apoptosis. (**A**) Quantification of EC viability 0–72 h post-infection with AdGFP, AdNull or AdN2ICD using a DNA content assay. (**B**) Apoptosis detection 72 h post-transduction after AnnexinV and Propidium Iodide (PI) staining. (**C**) PhiPhiLux™ system to detect real-time activation of caspase3/7 in live ECs after 48 h post-infection for 18 hours, A representative image after imaging is shown in panel C. Caspase3/7 positive cells/field (in %) were counted every 2 hours during the experiment (**D**). A western blot analysis for cleaved-forms of caspase-3, -7 and PARP in response to Notch2 NICD (**E**). (**F**) A colorimetric assay for caspase 3 and 7 activation 48 h after transduction.

Notch2 signaling was then tested for regulation of EC survival. First, the effect of N2ICD on EC viability was examined by a DNA content analysis from 24 h to 72 h after infection (Figure 2A). At 24 h, no significant effect was observed. At 48 h, a significant decrease in cell viability (46.5±5.9%) was found when ECs were infected with AdN2ICD at moi 40 while a lower dose of virus (moi 20) decreases cell survival by 31.8±8.4% indicating that N2ICD-induced cell death is a dose-dependent process. Finally, at 72 h post-infection, in contrast to AdGFP and AdNull controls that did not alter cell survival, AdN2ICD was sufficient to drastically induce cell death (40.0±14.0% and 67.5±6.1% of cell death for moi 20 and 40, respectively). To test whether EC death reflected apoptosis, ECs were labeled with AnnexinV and Propidium iodide 72 h after infection and apoptotic cells (AnnV+) were detected by flow cytometry. As shown in Figure 2B, N2ICD strongly induces apoptosis in a dose dependent manner. AdN2ICD transduced ECs displayed 58.2% and 77.5% of apoptotic cells for moi 20 and 40, respectively. In contrary, no effect was observed after AdGFP infection or with the corresponding empty Adenovirus at a moi of 40 thus attesting that the deleterious effect of AdN2ICD was not due to GFP nor to the viral charge. No necrotic cells (AnnV-PI+) were detected. To further characterize N2ICD-mediated apoptosis, we first used a PhiPhiLux™ system to detect real-time activation of caspase in live cells. PhiPhiLux incorporated into a cell is specifically cleaved by caspase-3 or caspase-7, and the cleaved products give fluorescence that can be detected by fluorescent Time lapse videomicroscopy imaging. After 48 h post-infection, ECs were subjected to time lapse imaging for 18 h (Figure 2C). Caspase3/7 positive cells/field (in %) were counted every 2 hours during the experiment (Figure 2D). This experimental setting allowed us to follow AdN2ICD-mediated apoptosis induction in reat time. As shown for AnnexinV staining, AdN2ICD was associated with a high number of caspase3/7-dependent apoptosis (37%) as compared to AdGFP and AdNull controls (5% and 2%, respectively) 66 h after infection.

Moreover, western blots were used to examine cleavage of caspase-3, -6, 7-9 and PARP in response to Notch2 NICD (Figure 2E). The 17-kDa form of cleaved caspase-3 was detected in EC overexpressing N2ICD at 48 and 72 h and parallels a decrease in full caspase-3 expression. Correlated to caspase-3 activation, clivage of caspase-7 and PARP were also observed. In contrast, no cleavage of caspase-6 and -9 was found (data not shown). To quantify caspase activation by N2ICD, we used a colorimetric assay for caspase-3 and -7 activation. As shown in Figure 2F, ECs overexpressing N2ICD exhibited a strong caspase activity as compared to AdGFP and AdNull controls (40.9 fold and 93.4 fold increase, respectively).

Protective effect of the pan caspase inhibitor Z-VAD-FMK in AdN2ICD-transduced ECs further confirmed that Notch2 mediates EC death by inducing caspase-mediated apoptosis (supplemental Figure S2).

**Figure 3:**
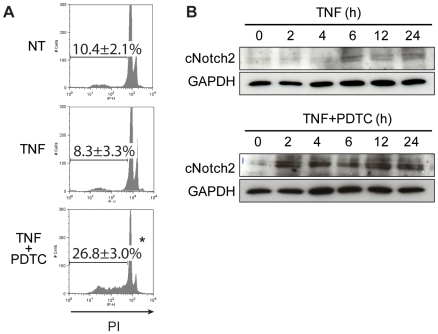
TNF and PDTC treatment induces EC mortality. EC death induction by DNA content assay (**A**) and activated cleaved-Notch2 expression by Western Blot (**B**) after TNF and PDTC treatment. Results are representative of 3 independent experiments. *p<0.05 versus controls.

In vascular ECs, TNF promotes EC activation/dysfunction but low levels of apoptosis, notably by inducing protective signaling pathways. To test the relevance of Notch2 induction and its pro-apoptotic function in ECs, we test its regulation in a TNF-related apoptosis model. First, we showed in Figure 3A that EC apoptosis can be triggered by using the combination of TNF and the antioxidant pyrrolidine dithiocarbamate (PDTC), also commonly used as a non specific NFκB inhibitor. Interestingly, as TNF fail to induce EC apoptosis and correlates with a transient Notch2 activation after 6 h of treatment, the apoptotic combination of TNF and PDTC was associated with a stronger and sustained activation of Notch2 receptor between 2 h and 24 h of treatment (Figure 3B) thus confirming the implication of Notch2 signaling in EC apoptosis process.

Altogether, these results suggest that a major consequence of Notch2 activation in vascular ECs is the induction of apoptosis in a time- and dose-dependent manner.

### Activated Notch2 Broadly Represses the Transcription of Apoptosis Mediators

**Figure 4:**
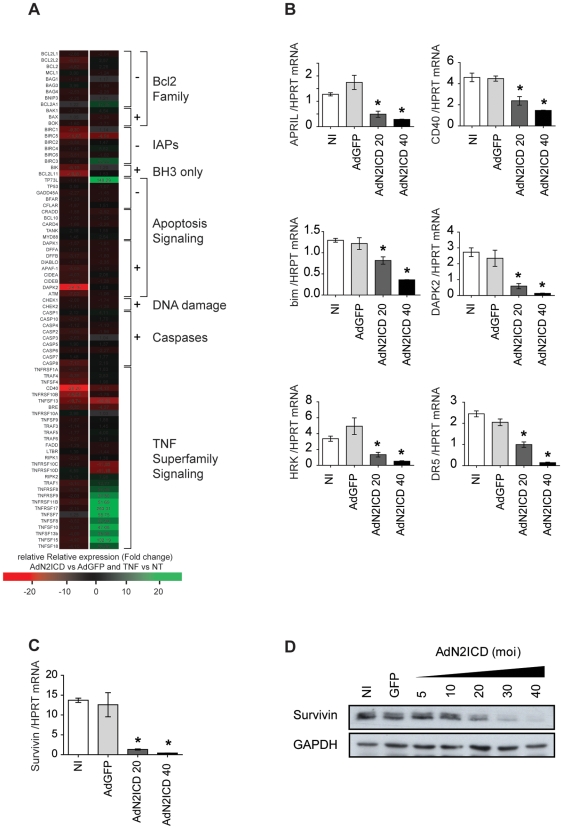
Notch2 signaling downregulates apoptosis mediators. (**A**) A schematic representation of apoptosis-related transcripts repressed (red) or induced (green) by N2ICD or TNF determined by a dedicated PCR array. Means are shown as fold changes compared to AdGFP or untreated ECs, respectively. (**B**) Validation of transcripts regulation by qRT-PCR in ECs transduced with AdN2NICD (moi 20 and 40) in comparison to controls (non infected (NI) and AdGFP) (**B–C**). (**D**) Dose-response effect of AdN2ICD on Survivin protein expression by western blot. Results are representative of 5 independent experiments. *p<0.05 versus controls.

To examine the underlying mechanisms by which Notch2 NICD sensitizes ECs to apoptosis, an apoptosis-dedicated qRT-PCR array displaying 88 pro- and anti-apoptotic molecules was used. Fold changes in gene expression between AdN2ICD- and AdGFP-transduced ECs are depicted in the Figure 4A, as well as TNF-treated *versus* non-treated ECs as control. For the selection of candidate genes, only transcript exhibiting an over 10-fold ratio regulation compared to the AdGFP control were considered. Consequently, 7 candidates were selected; all were down regulated in response to N2ICD. We found that 5 out of 7 regulated genes were the pro-apoptotic molecules Bim, death-associated protein kinase 2 DAPK2, HRK (Harakiri) and DR5 (a TRAIL receptor) and CD40 whose expression was highly repressed in response to N2ICD (13.5-, 24.8-, 10.9-, 14.1-, 28.2-fold decrease *versus* AdGFP control, respectively; *p<0.05) (Figure 4A). Our findings also indicate that N2ICD significantly decreases gene transcript levels for 2 protective molecules: survivin and April (11.9- and 10.7-fold decreased as compared to control, respectively; *p<0.05). Decreased transcript levels were further confirmed by qRT-PCR and found correlated to Notch2 expression (Figure 4B).

Since we found that constitutive expression of N2ICD in cultured ECs elicits apoptosis but also impairs EC proliferation (supplemental Figure S3), we speculate that survivin, a potent regulator of both functions, notably in ECs, also found inhibited by TNF, could be a key player of Notch2 signaling [20], [21]. Therefore, we further examined the possible involvement of survivin in Notch2-mediated apoptosis. First, Figure 4C confirms that N2ICD drastically repressed survivin transcript level with a dose-dependent effect (9.6±1.0- and 30.8±1.3-fold decrease at moi 20 and moi 40, respectively; *p<0.05). Moreover, regulation of survivin protein expression by Notch2 was confirmed by western blotting and was found dependent on N2ICD in ECs. Our results suggest therefore that Notch2 activation could mediate EC apoptosis by negatively regulating the key protective *survivin* gene (Figure 4D).

### Notch2 Silencing by RNA Interference Promotes Survivin Expression and Rescues ECs from Apoptosis

**Figure 5:**
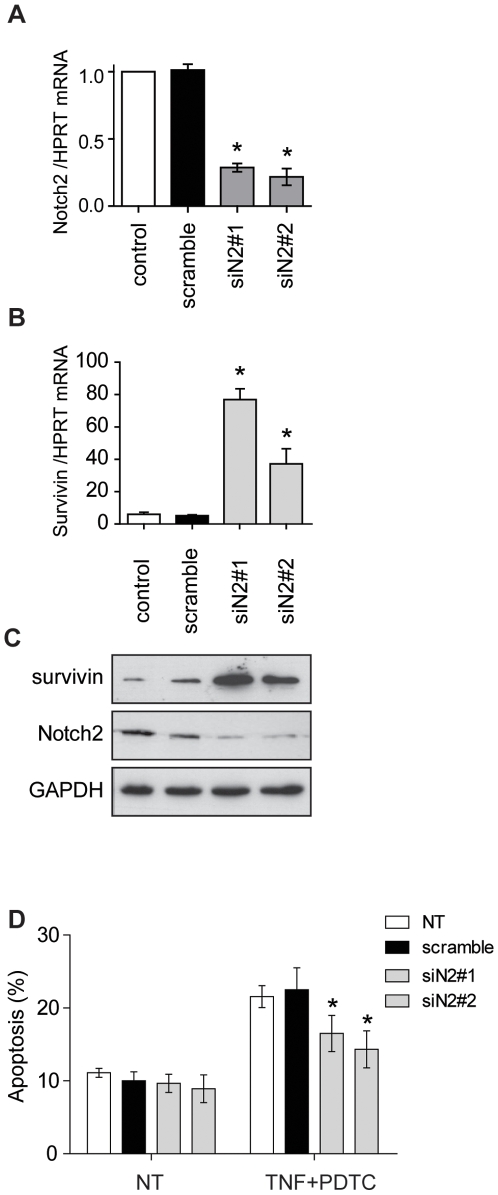
Notch2 knockdown increases survivin and rescues ECs from apoptosis. (**A**) Validation of Notch2 silencing in ECs by qRT-PCR. (**B**) qRT-PCR for survivin mRNA in ECs transfected with a scramble siRNA or siRNAs targeting Notch2. (**C**) Survivin and Notch2 expression by western blot. (**D**) DNA content analysis of siRNA transfected ECs after induction of apoptosis by TNF and PDTC (NT: non transfected cells). Results are means±SEM of 3 independent experiments.*p<0.05 *versus* scramble.

Based on the above results, we further investigated the possible crosstalk between apoptotic pathway and Notch2 signaling in ECs. Because many genetic studies showed that the Notch pathway is very dose-sensitive, we used a loss-of-function model for Notch2 using Notch2 siRNA. This approach was designed to determine the function of endogenous Notch2 signaling on EC survival. To test whether inhibition of basal Notch2 could modulate apoptosis, ECs were transfected with specific Notch2 targeted siRNAs. Non targeting scramble siRNAs were used as controls. Among the 3 tested siRNAs, 2 displayed a significant down-regulation of Notch2, at mRNA level by qRT-PCR (71.2±3.4% 78.6±6.1% of inhibition for siN2#1 and #2, respectively, as compared to control siRNA) (Figure 5A). Figure 5B demonstrates that, in contrast to N2ICD that inhibited survivin expression, Notch2 silencing significantly increases survivin mRNA in vascular ECs (9.8- and 7.7-fold increase *versus* scramble control). This regulatory effect was further confirmed at protein level by western blotting (Figure 5C).

To test whether Notch2 inhibition could protect from apoptosis, ECs were cultured for 48 h after siRNA transfection before induction of apoptosis by TNF and PDTC treatment (supplemental Figure S4). Cell death was then quantified by DNA content analysis (Figure 5D). First, we observed that knockdown of Notch2 did not modify basal EC viability. Moreover, when EC apoptosis is induced by a 24 h treatment with TNF and PDTC, Notch2 knockdown significantly rescued ECs from apoptosis as compared to scramble control.

Altogether, these findings suggest that survivin regulation is controlled at the Notch level and that Notch2 inhibition protects ECs from apoptosis by increasing survivin expression level.

### Notch2-Mediated Endothelial Apoptosis Is Dependent on Survivin Down-Regulation

**Figure 6:**
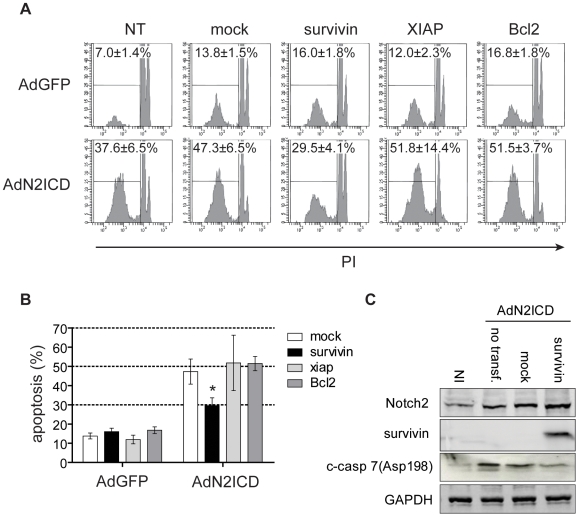
Survivin prevents Notch2-mediated apoptosis. ECs were transduced or not (NT) with AdGFP (5 moi) or AdN2ICD (30 moi) after transfection with a plasmid encoding survivin, XIAP or Bcl2 full cDNAs or the empty vector (mock) as a control. Cell death was measured by DNA content (**A**). Corresponding means±SEM of 7 independent experiments (**B**). *p<0.05 *versus* mock. A representative western blotting analysis for Notch2, survivin and cleaved-caspase 7 expression in ECs non infected (NI) or transduced with AdN2ICD (30 moi) 24 h prior to transfection with mock or survivin plasmids. Cell lysates were collected 48 h after infection (representative of 3 independent experiments).

To validate the direct implication of survivin in Notch2-mediated apoptotic functions, we transfected ECs with survivin, XIAP (a close member of the survivin IAP family) or Bcl2 cDNA before N2ICD overexpression. Considering a transfection efficiency of 40% in HUVEC with plasmids, ectopic expression of survivin very efficiently prevents cell apoptosis mediated by N2ICD (29.5±4.1% *versus* 47.3±6.5% of cell death for Null control). In contrast, no protective effect was observed with XIAP nor Bcl2, attesting the specific involvement of survivin in Notch2-mediated apoptosis in ECs (Figure 6A–B). Because survivin may directly bind and inhibit caspases, we tested by western blotting whether survivin decrease in response to Notch2 activation was implicated in N2ICD-dependent caspase activation. As shown in Figure 6C, ectopic expression of survivin in N2ICD overexpressing ECs was sufficient to significantly reduce N2ICD-mediated caspase 7 activation. These results therefore suggest that N2ICD triggers caspase-dependent apoptosis by decreasing survivin anti-caspase activity.

## Discussion

Here, we provide evidence for a novel link between Notch and TNF signaling, where Notch2 is activated in response to TNF and directly controls expression of several genes involved in EC survival and apoptosis. Activation of Notch2 leads to a rapid decrease in survivin mRNA and protein expression, and survivin upregulation was obtained by the selective knockdown of Notch2 in ECs, indicating that survivin regulation is controlled at the Notch level.

Overall, our study reports a pro-apoptotic role for Notch2 signaling and indicates that specific Notch receptors control distinct cell fate decisions in the vascular system. The major vascular defects observed in Notch1^−/−^, exacerbated in Notch1^−/−^ Notch4^−/−^ mutant animals and the restricted expression of Notch4 in ECs led studies to focus on these Notch receptors in ECs. Notch1 and Notch4 seems to share common major functions in angiogenesis as both protect ECs from apoptosis and decrease growth rate [9], [22], [23], [24], [25], [26]. These findings also illustrate possible compensatory mechanisms in Notch4^−/−^ mice by Notch1 which do not develop severe vascular defects.

Nonetheless, little is known about Notch2 implication in vascular homeostasis. We show here that Notch2 exerts opposite effects on EC survival as compared to Notch1 and Notch4. This pro-apoptotic function has already been reported in breast and skin cancers [27], [28]. However, controversial results in various cancer models seem to point out cell-type or cellular context specific orientation of Notch2 apoptotic function [29], [30], [31], [32].

Our findings further substantiated the emerging concept that the Notch molecules display unique functions. This functional specificity was already exemplified in the lymphopoietic system by the genetic mouse models in which Notch1 deficiency results in impaired T-cell development [33], while Notch2 deficiency causes a dramatic absence of MZB cells [34]. Accordingly, a recent study demonstrates that activation of different Notch receptors in the human mammary adenocarcinoma cell line MDA-MB-231 drives dramatically opposing effects, leading to either increased apoptosis in the case of Notch2 or increased proliferation in the case of Notch4 [35]. This is also in line with the finding that different Notch ICDs have different target sequence selectivity [36]

Strikingly, it remains a critical question how various mammalian Notch receptors and ligands exert their unique functional activities. Indeed, such specificity is not completely explained by differential expression patterns or preferential molecular interactions of Notch receptors or ligands, suggesting that other genes can contribute to specifying Notch receptor functions. Our previous study [13] and results presented here indicate that TNF elicits a drastic change in the pattern of Notch receptors expressed on vascular ECs. This change corresponds to phenotypic switch where Notch4 is replaced by Notch2. No significant change in Notch1 protein was observed. Among Notch ligands, only Dll1 was found to be upregulated by inflammatory cytokines (data not shown), providing a potential mechanism for Notch2 activation in response to TNF. Functionally, activation of Notch2 signaling favors cell death while Notch4 activation was shown to be protective [9]. Thus it is tempting to speculate that cytokines (i.e. TNF) by modulating Notch signaling and function provide another step to Notch regulation. Another (non exclusive) possibility to consider comes from the recent study by Wu *et al.* that discovered an unexpected role for the Notch coactivator *Maml1* in mediating the specific signaling of Notch2 [37]. These findings suggesting that individual *Maml* coactivators may regulate molecular specificity of Notch receptor functions remains to be confirmed.

Apoptosis is believed to be an important factor in vascular remodeling in normal and pathologic conditions [19]. Survivin is an inhibitor of apoptosis protein (IAP) that is upregulated in cancer and has recently been implicated in vascular injury [38]. Survivin was originally detected in tumors; however, subsequent studies have revealed that many normal adult tissues express survivin albeit at levels lower than cancer cells. Survivin is an essential protein in that disruption of the survivin locus in mice results in early embryonic lethality [39]. This reflects a critical dual function of survivin in the regulation of cell division and the preservation of cell viability. The demonstration that survivin levels in normal tissues can be up-regulated by cytokines suggests that survivin may have physiologic roles in regulating proliferation and survival [40]. Although the existence of functional survivin–caspase complexes is controversial, the ability of survivin to inhibit apoptosis has been clearly demonstrated [41]. Expression of survivin protects normal or transformed cells from apoptosis [42] while forced expression of survivin inhibits various forms of cell death both *in vitro* and *in vivo* [39]. Lack of endothelial cell survivin resulted in embryonic lethality. Mutant embryos had prominent and diffuse haemorrhages from embryonic day 9.5 (E9.5) and died before E13.5 [43], pointing out a key protective role of survivin in ECs. Our findings are complementary to studies in primary cultured human ECs and VSMCs where induction of apoptosis by TNF and cycloheximide or inflammatory cytokines (IFNγ, TNF, IL1β) was completely prevented by survivin overexpression [21], [44]. Moreover, whether survivin inhibition by Notch2 could be implicated in proliferation blockade resulting from Notch2 induction remains to be verified. Nonetheless, survivin may act upstream of gene expression and directly influence the transcription of pivotal growth-related gene(s) in vascular cells. In the future, this information might lead to novel strategies to prevent cancer and vascular diseases where Notch/survivin signaling plays a pathogenic role.

In conclusion, we show for the first time that TNF signaling strongly upregulates and activates Notch2 in vascular ECs. Our findings further indicate that dysregulated Notch2 signaling sensitizes vascular ECs to apoptosis and demonstrate a major role for survivin as effector of Notch signaling.

## Supporting Information

Figure S1 Activation of Notch2 signaling in ECs.(A) Dose-dependent transduction of ECs according to moi for the recombinant AdGFP and AdN2NICD. Results are expressed as percentage of GFP-expressing cells determined by flow cytometry 24h post-infection. (B) GFP expression analysis by fluorescence microscopy in ECs transduced with AdGFP or AdN2ICD. (C) Western blot for Notch2 and GAPDH expression. Non-infected (NI) and ECs infected with AdGFP are used as controls. (D) Immunoreactivity for N2ICD correlated to GFP expression in nuclei of ECs transduced with AdN2ICD. (E) Cotransfection of ECs with a CBF1/luciferase reporter plasmid and a plasmid encoding N2ICD. Controls included non transfected ECs (NT), CBF1/luc-transfected cells treated with medium or DAPT. Results are means of 4 independent experiments (arbitrary units).
(3.78 MB TIF)

Figure S2 AdN2ICD-mediated apoptosis protection by caspase inhibition. Non infected (NI), AdGFP and AdN2ICD-transduced HAECs were cultured with or without the pan caspase inhibitor zvad for 48h. Cell death was measured by DNA content assay. Results are representative of 3 independent experiments.
(1.39 MB TIF)

Figure S3 Notch2 inhibits EC proliferation in response to FGF. Transduced ECs by AdN2ICD (moi 20 and 40) or AdGFP control vector (moi 5) proliferation in response to FGF was tested by tritiated-thymidine uptake. Results are means ± SEM from 3 independent experiments. *p<0.05 vs AdGFP control.
(0.68 MB TIF)

Figure S4 Induction of EC apoptosis by TNF and PDTC treatment. ECs were treated with TNF and PDTC for 24 h (NT: non treated cells). Apoptosis was quantified by flow cytometry after Annexin V and Propidium Iodide (PI) labeling. A representative dot plot (A) and graphic representation (B) are shown. Results are means ± SEM from 3 independent experiments. *p<0.05 vs untreated cells.
(1.01 MB TIF)
