# Supplementary figures and images for: Notch2 Signaling Sensitizes Endothelial Cells to Apoptosis by Negatively Regulating the Key Protective Molecule Survivin
Source: PLoS One. 2009 Dec 11;4(12):e8244. doi: 10.1371/journal.pone.0008244 (PMC2785888; doi:10.1371/journal.pone.0008244)

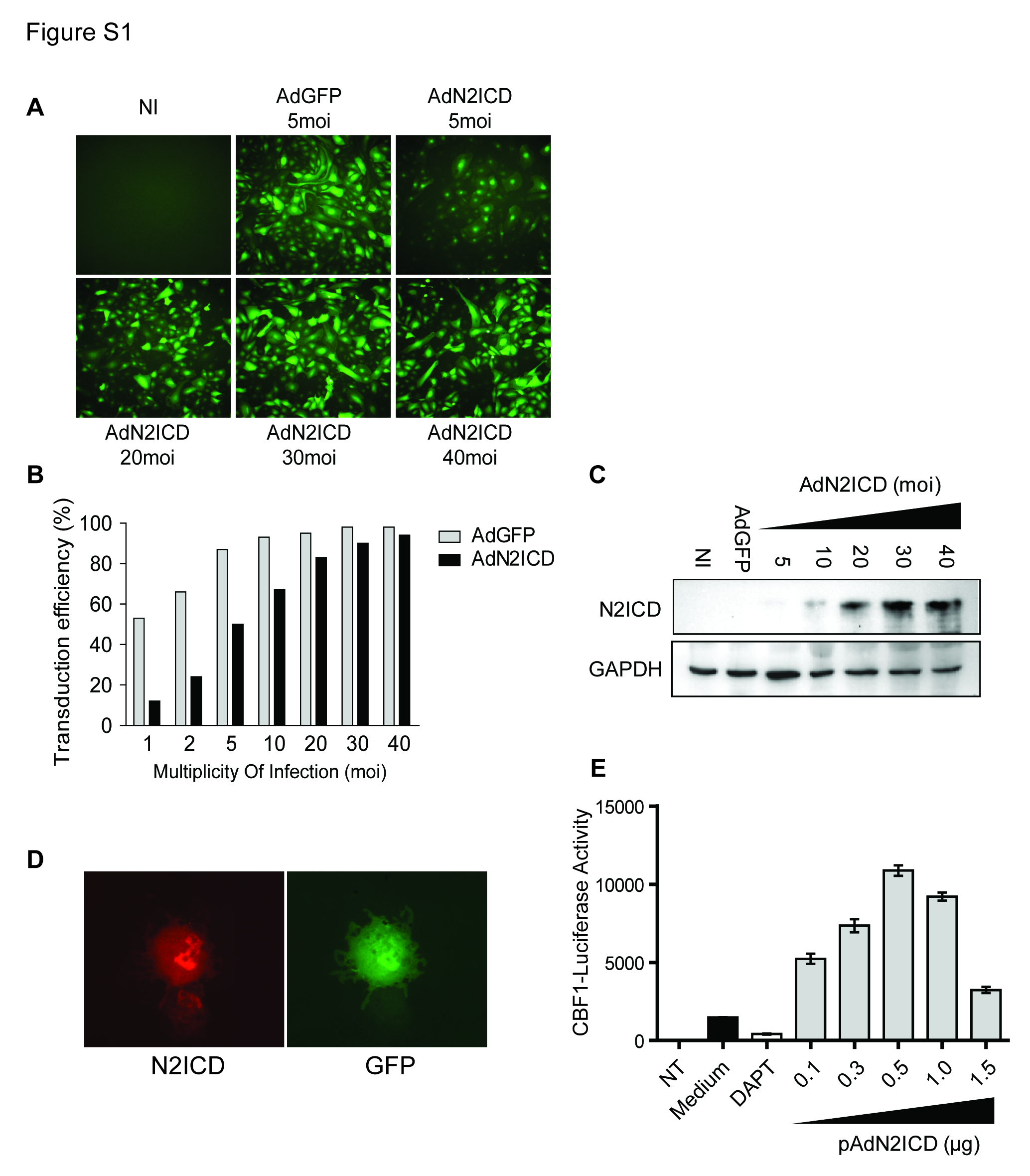

Supplement: Figure S1 — Activation of Notch2 signaling in ECs.(A) Dose-dependent transduction of ECs according to moi for the recombinant AdGFP and AdN2NICD. Results are expressed as percentage of GFP-expressing cells determined by flow cytometry 24h post-infection. (B) GFP expression analysis by fluorescence microscopy in ECs transduced with AdGFP or AdN2ICD. (C) Western blot for Notch2 and GAPDH expression. Non-infected (NI) and ECs infected with AdGFP are used as controls. (D) Immunoreactivity for N2ICD correlated to GFP expression in nuclei of ECs transduced with AdN2ICD. (E) Cotransfection of ECs with a CBF1/luciferase reporter plasmid and a plasmid encoding N2ICD. Controls included non transfected ECs (NT), CBF1/luc-transfected cells treated with medium or DAPT. Results are means of 4 independent experiments (arbitrary units). (3.78 MB TIF) [file pone.0008244.s001.tif]

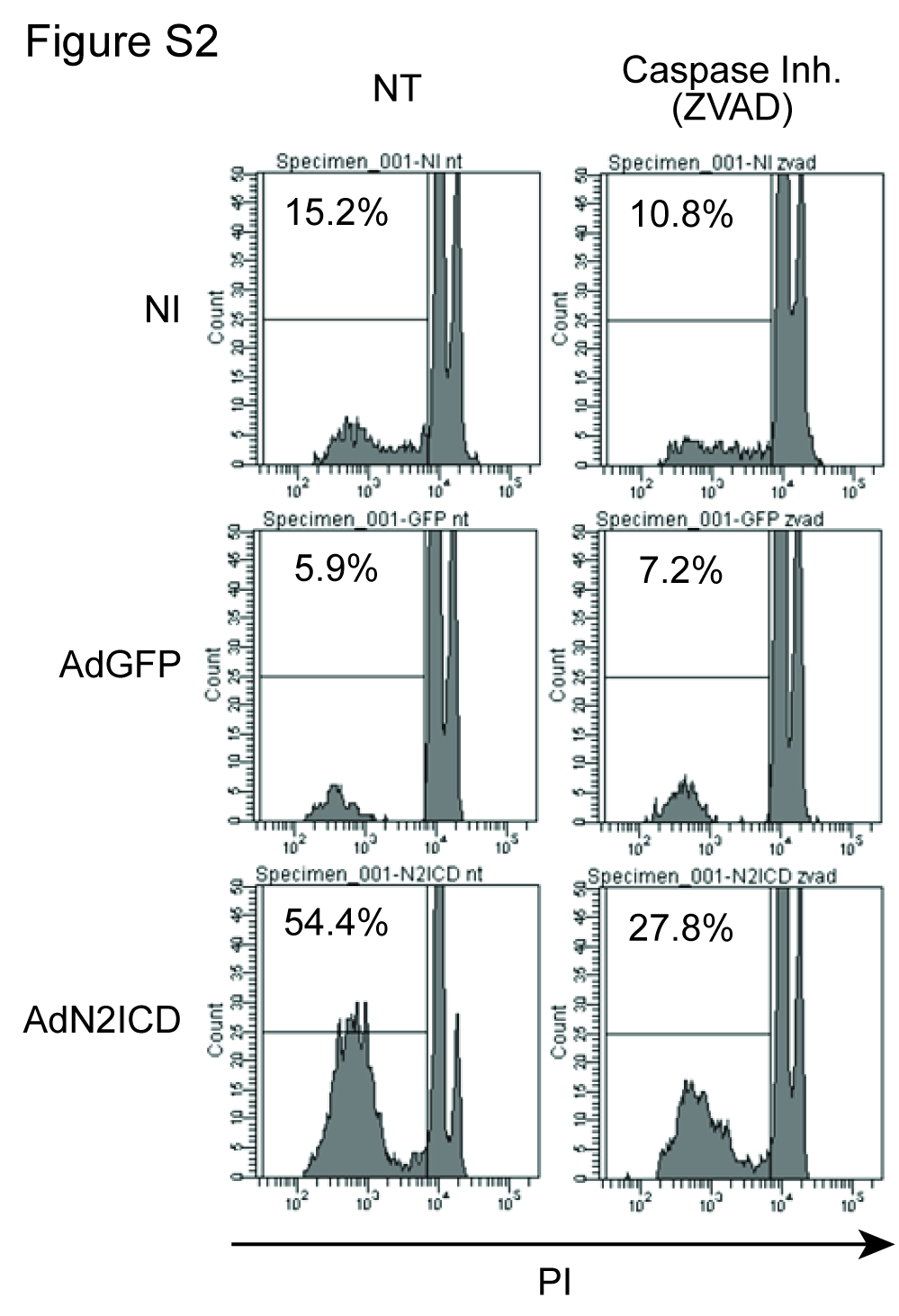

Supplement: Figure S2 — AdN2ICD-mediated apoptosis protection by caspase inhibition. Non infected (NI), AdGFP and AdN2ICD-transduced HAECs were cultured with or without the pan caspase inhibitor zvad for 48h. Cell death was measured by DNA content assay. Results are representative of 3 independent experiments. (1.39 MB TIF) [file pone.0008244.s002.tif]

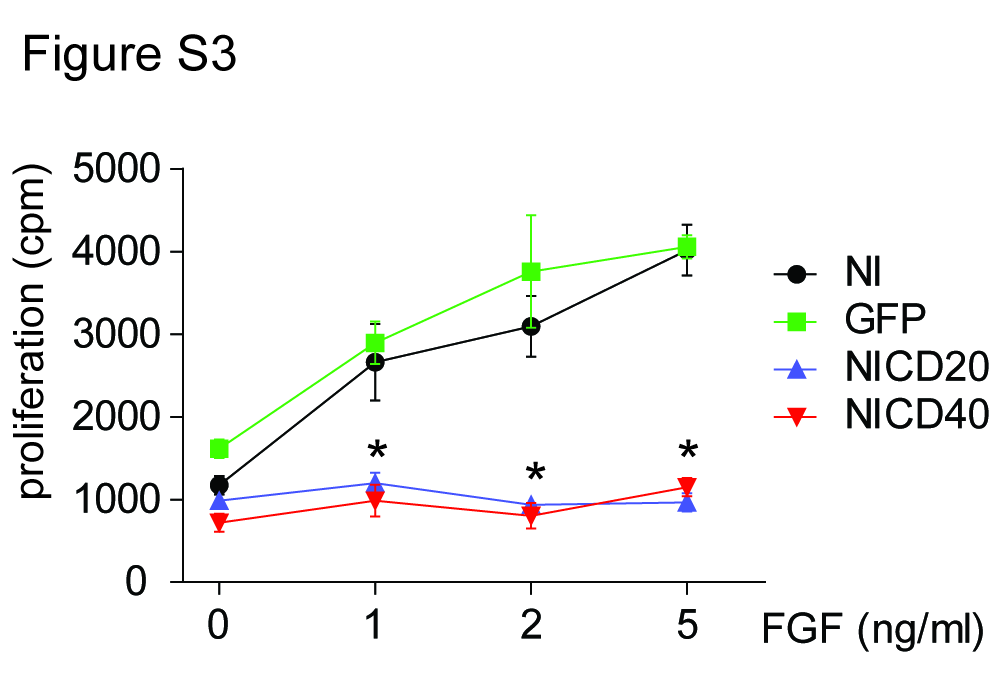

Supplement: Figure S3 — Notch2 inhibits EC proliferation in response to FGF. Transduced ECs by AdN2ICD (moi 20 and 40) or AdGFP control vector (moi 5) proliferation in response to FGF was tested by tritiated-thymidine uptake. Results are means ± SEM from 3 independent experiments. *p<0.05 vs AdGFP control. (0.68 MB TIF) [file pone.0008244.s003.tif]

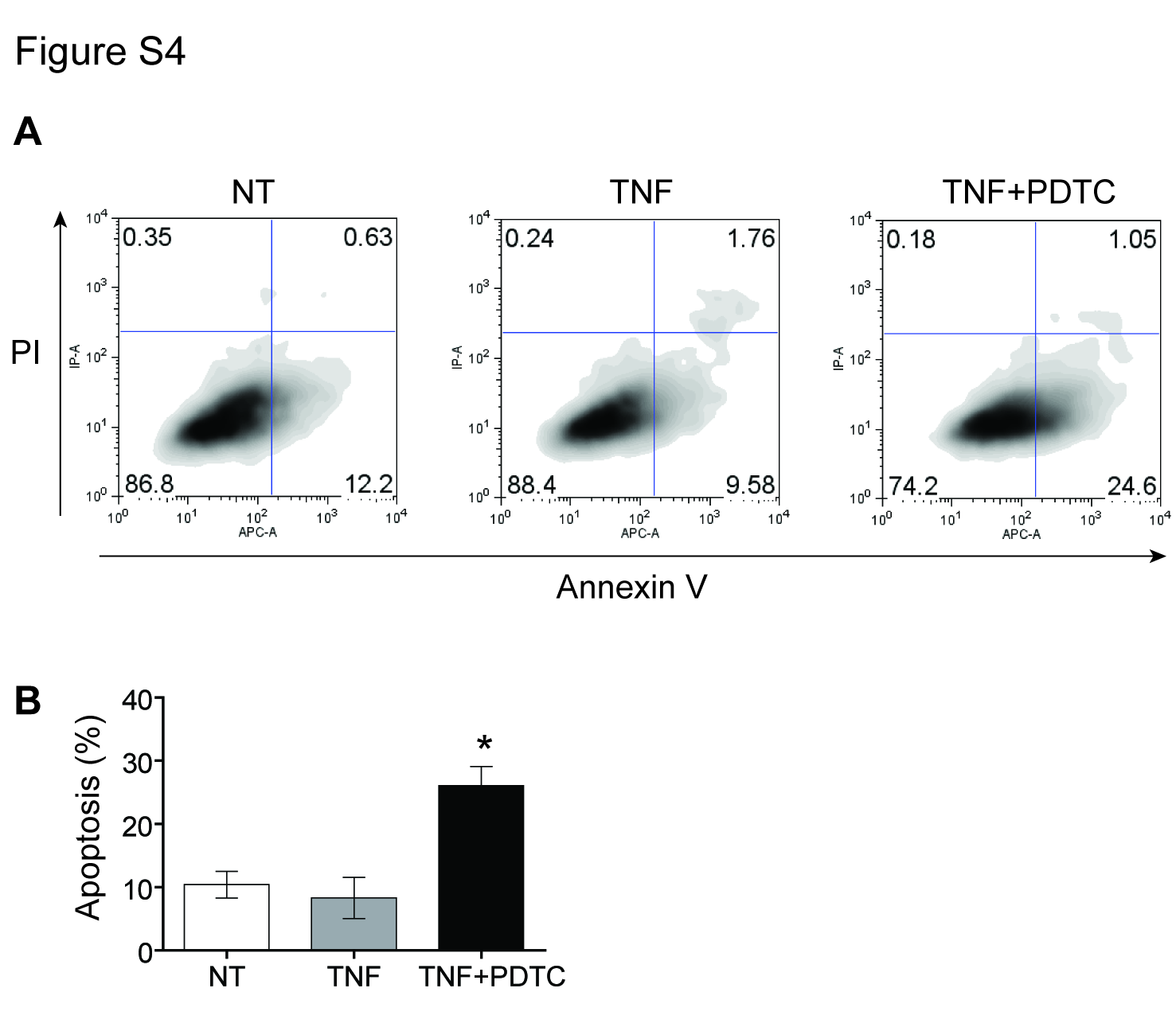

Supplement: Figure S4 — Induction of EC apoptosis by TNF and PDTC treatment. ECs were treated with TNF and PDTC for 24 h (NT: non treated cells). Apoptosis was quantified by flow cytometry after Annexin V and Propidium Iodide (PI) labeling. A representative dot plot (A) and graphic representation (B) are shown. Results are means ± SEM from 3 independent experiments. *p<0.05 vs untreated cells. (1.01 MB TIF) [file pone.0008244.s004.tif]
